# *Aeromonas sobria* necrotizing fasciitis and sepsis in an immunocompromised patient: a case report and review of the literature

**DOI:** 10.1186/1752-1947-8-315

**Published:** 2014-09-22

**Authors:** Savino Spadaro, Angela Berselli, Elisabetta Marangoni, Anna Romanello, Maria Vittoria Colamussi, Riccardo Ragazzi, Silvia Zardi, Carlo Alberto Volta

**Affiliations:** 1 Department of Morphology, Surgery and Experimental Medicine, Sant’ Anna Hospital, University of Ferrara, Via Aldo Moro 8, Ferrara, FE 44124, Italy

**Keywords:** *Aeromonas sobria*, Immunocompromised host, Necrotizing fasciitis, Sepsis

## Abstract

**Introduction:**

*Aeromonas veronii* biovar sobria is a rare cause of bacteremia, with several studies indicating that this isolate may be of particular clinical significance since it is enterotoxin producing. A wide spectrum of infections has been associated with *Aeromonas* species in developing countries that include gastroenteritis, wound infections, septicemia and lung infections. This infection, caused by *Aeromonas* species*,* is usually more severe in immunocompromised than immunocompetent individuals. We here describe a case of soft tissue infection and severe sepsis due to *Aeromonas sobria* in an immunocompromised patient.

**Case presentation:**

A 74-year-old Caucasian man with a clinical history of chronic lymphocytic leukemia and immune thrombocytopenia, periodically treated with steroids, was admitted to our Intensive Care Unit because of necrotizing fasciitis and multiorgan failure due to *Aeromonas sobria*, which resulted in his death. The unfortunate coexistence of a *Candida albicans* infection played a key role in the clinical course.

**Conclusion:**

Our experience suggests that early recognition and aggressive medical and surgical therapy are determinants in the treatment of severe septicemia caused by an *Aeromonas sobria* in an immunocompromised patient.

## Introduction

Necrotizing fasciitis is a destructive soft tissue infection characterized by an extensive necrosis in skin, subcutaneous tissues and fascia that is associated with systemic toxicity and fulminant course with a mortality rate varying from 40% to 60% [1]. Immunosuppression, diabetes mellitus, alcoholism, end-stage renal disease, malignancy and chemotherapy have all been suggested as predisposing factors in necrotizing fasciitis [2].

Bacteria associated with necrotizing fasciitis include *Aeromonas* species [3]. *Aeromonas* organisms are Gram-negative small bacilli isolated from a variety of environmental sources including water, seafood, meat and vegetables, with the ability to colonize both humans and animals [4]. The clinical spectrum of *Aeromonas* species infection in humans includes acute gastroenteritis, hepatobiliary tract infection, pneumonia, empyema, meningitis, septic arthritis, osteomyelitis, endocarditis, bacteremia, burn and wound infection [5,6].

Clinical isolates in 85% of human infections involve three phenotypically defined species: *Aeromonas hydrophila, Aeromonas caviae* and *Aeromonas veronii* biovar sobria [7]. *A. veronii* biovar sobria is predominantly isolated in patient’s blood and is more pathogenic than *A. hydrophila*[7,8]. The possible portals of entry for *Aeromonas* bacteremia are gastrointestinal tracts, skin lesions, previous surgery or local trauma in an aqueous environment [9]. After adhesion to epithelial cells, *Aeromonas* produces many virulent factors which destroy host epithelial barriers and impair immune cells, including exoenzymes, cytotoxic and cytotonic enterotoxins, hemolysins, proteinases, lipases, agglutinins, various hydrolytic enzymes, translocation capacity and a *Aeromonas sobria* cytotoxic factor [4,10,11]. *Aeromonas* infections can develop in healthy and trauma patients, but immunocompromised hosts with hematologic malignancy, cancer and hepatobiliary diseases are considered to be at greatest risk [12]. Patients with severe wound infection (myonecrosis) caused by this microorganism also develop sepsis, and 90% of patients succumb to their infections [13]. The fatality rate of *Aeromonas* soft tissue infections and bacteremia is high and reportedly ranges from as much as 28% to 73%, with septic shock being the cause of death in the majority of patients [14].

## Case presentation

We present the case of a 74-year-old Caucasian man with a medical history of ischemic heart disease, atrial flutter, arterial hypertension, severe aortic stenosis, chronic lymphocytic leukemia and related immune thrombocytopenia periodically treated with steroids (every 28 days). He was referred to our Emergency Room for fever and an altered state of consciousness. During the clinical examination, his temperature was 40°C, his blood pressure was 120/80mmHg, pulse 89 beats per minute, arrhythmic and his initial oxygen saturation checked by pulse oximetry was 95% in room air. He was somnolent but easily aroused and on examination presented pulmonary bibasal crepitations, heart murmur and a hematoma on his left foot. A laboratory evaluation revealed an increase in white blood cell (WBC) count (15.0×10^3^/μL; reference value 4.00 to 11.00×10^3^/μL), platelet count of 56×10^3^ cells/μL (reference value 150 to 450×10^3^ cells/μL), and C-reactive protein 6.6mg/dL (reference value 0.0 to 0.5mg/dL). Four days before hospital admission, while fishing, his left hand had been scratched by the dorsal fin spine of a black bullhead (*Ameiurus melas*, catfish); no physical signs *in situ* were present on initial examination. He was hospitalized in the Clinic of Infectious Diseases, Ferrara. On admission, an empiric antibiotic treatment with ampicillin-sulbactam (3g every 8 hours, intravenous) was initiated intravenously; blood cultures were performed. The day after admission, his vital signs and body temperature were normal but he began to complain of muscle weakness and of severe pain in his lower extremities, radiating to his knees. A neurological examination revealed an asymmetric hyposthenia and hypoesthesia of his lower limbs, bilateral areflexia of his Achilles tendon, lower extremity acute paresis, and bladder dysfunction. A spine magnetic resonance imaging excluded cauda equina syndrome, while the vascular surgeon excluded an acute ischemic peripheral event. To exclude endocarditis an echocardiography was performed, revealing a global cardiac contractile dysfunction with a severe reduction in ejection fraction (35%). Antibiotic therapy was changed to ceftriaxone (2g every 24 hours, intravenous) and levofloxacin (500mg every 12 hours, intravenous) because of the further increase in his WBC count (24.3×10^3^ cells/mm^3^), and blood cultures revealed an increase in Gram-negative bacilli that were later confirmed to be *A. sobria,* the latter being identified by Vitek® II method.

**Figure 1:**
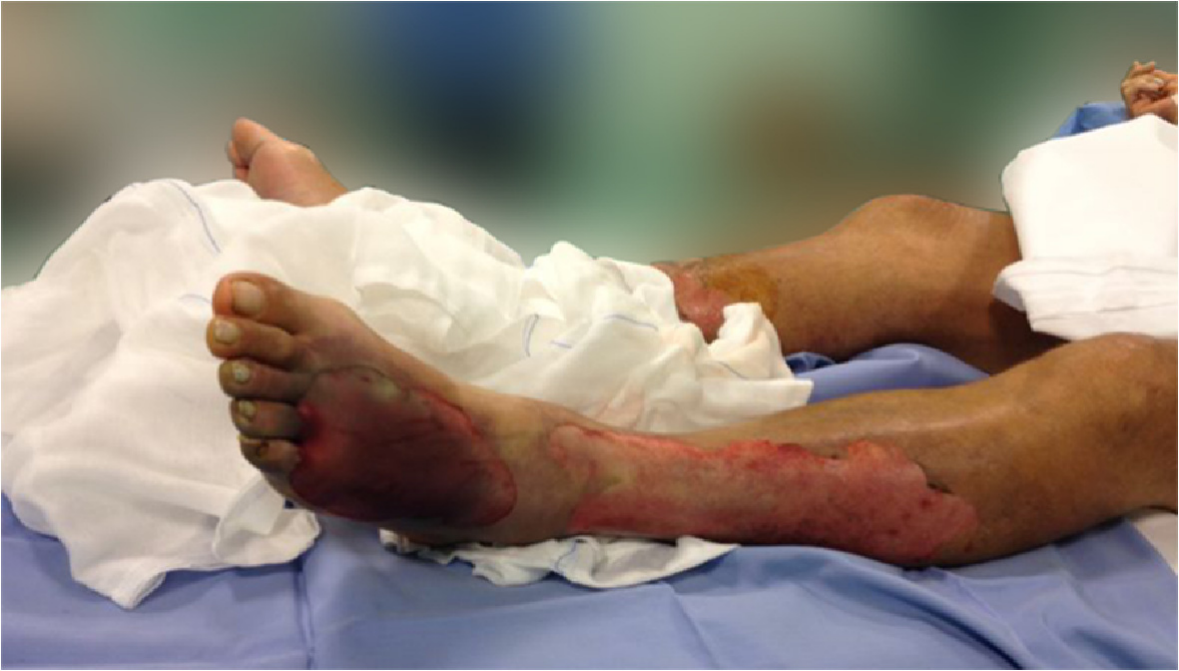
Early skin lesions of lower limbs before fasciotomy.

**Figure 2:**
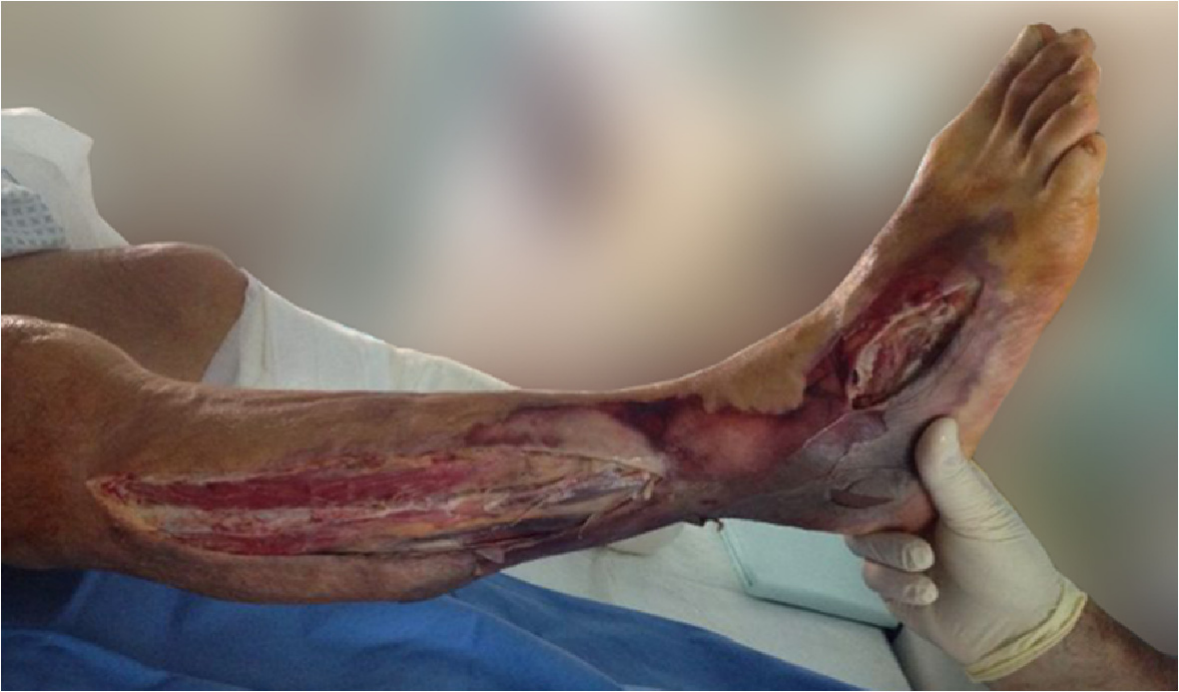
Gangrenous lower limbs after fasciotomy.

After 48 hours, his clinical condition deteriorated and he developed severe sepsis with hypotension (systolic blood pressure was 80mmHg) and peripheral vasoconstriction, acute renal failure with oliguria (urea: 159mg/dL and creatinine: 2.55mg/dL), and severe metabolic acidosis. He was therefore transferred to our Intensive Care Unit (ICU). At this time, laboratory values were as follows: WBC count of 17.4×10^3^ cells/mm^3^, platelet count of 38×10^3^ cells/mm^3^, procalcitonin 99.3ng/mL (reference value <0.05ng/mL), and C-reactive protein 46.9mg/dL (reference value 0 to 0.5mg/dL). An antimicrobial regimen was promptly implemented with meropenem (1g every 12 hours, intravenous) and linezolid (600mg every 12 hours, intravenous). Laboratory data suggested a diagnosis of rhabdomyolysis (creatinine phosphinase 5013U/L; reference value <190U/L), myoglobinemia 13270ng/mL (reference value 28 to 72ng/mL), lactate dehydrogenase 673U/L (reference value 240 to 480U/L) and, on physical examination, his lower limbs were warm in presence of erythema. After 12 hours, the swelling of his legs and progression of skin lesions (circumferential erythema developed in bullae formations) increased until a compartment syndrome developed, rapidly requiring surgical treatment with bilateral fasciotomy (Figures 1 and 2).

Blood culture results were available 3 days after ICU admission. The strain was identified as *Aeromonas sobria*. The isolate was susceptible to amikacin, cefepime, cefotaxime, ceftazidime, levofloxacin, ertapenem, gentamicin, and piperacillin-tazobactam. Resistance to meropenem and imipenem were also observed. Antibiotics were adjusted in relation to blood culture and sensitivities. Consequently, meropenem was discontinued and he was given ceftazidime (2g every 8 hours, intravenous).

**Figure 3:**
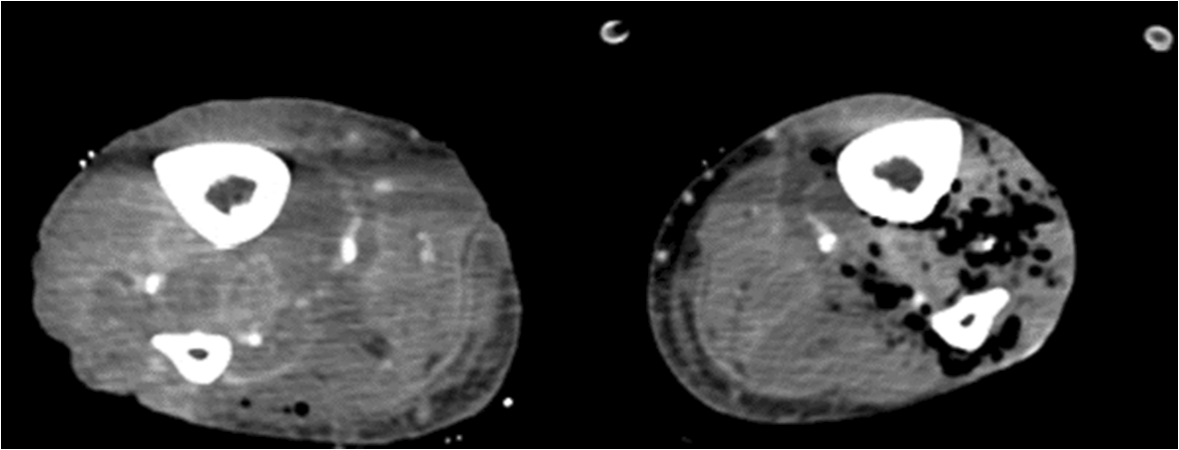
Computed tomography scans of legs demonstrating gas gangrene.

On ICU day 7, due to return of spiking fever and leg pain, computed tomography (CT) scans were performed. CT scans of his lower extremities showed progression to overt fasciitis with gaseous infiltration in the soft tissues (Figure 3). In view of the CT scan images and microbiological results, the surgeon was consulted and amputation for necrotizing infections of the extremities was performed. Deep wound cultures yielded growth of Gram-negative bacilli, which were identified as *A. sobria*. No anaerobes were detected from necrotic tissue. After surgical and medical treatment, his clinical status showed progressive improvement in renal, cardiocirculatory, splanchnic and metabolic function. Laboratory findings revealed no further signs of rhabdomyolysis (creatinine phosphinase 97U/L, lactate dehydrogenase 378U/L); a progressive reduction in WBC count (13.7×10^3^ cells/mm^3^), a platelet count of 122×10^3^ cells/mm^3^, procalcitonin 0.88ng/mL, urea 58mg/dL and creatinine 0.78mg/dL were observed. Over the course of weeks, consecutive blood cultures showed *Aeromonas* microorganisms were no longer isolated.

Although surgical wounds following bilateral amputation presented no further signs of necrosis, frequent surgical debridements were still necessary. In order to perform these painful procedures he was heavily sedated and required mechanical ventilation, which prolonged his stay in the ICU. Despite the aggressive treatment and administration of broad-spectrum antibiotics, including antifungal prophylaxis, the coexistence of immunocompromised host and comorbidities induced during his ICU stay, caused the development of an invasive candidemia (*Candida albicans*) which was ultimately responsible for his death.

## Discussion

The clinical manifestations and outcomes of *Aeromonas*-associated soft tissue infections vary in relation to the immune status of the host. Since *Aeromonas* species were considered rare pathogens of necrotizing fasciitis in immunocompromised patients [11,12], we present an uncommon case of *A. sobria* necrotizing fasciitis in Italy. In immunocompromised hosts, *Aeromonas*-associated soft tissue infections can be fulminant and fatal. Previous studies have shown that *Aeromonas* species infections are strongly associated with mortality [15]. Table 1 shows cases of *Aeromonas* species necrotizing fasciitis that have been reported in the literature. Similar features are revealed in the literature and previously described case reports have comparable clinical characteristics. Chang *et al.*[6] illustrated a fulminant necrotizing fasciitis caused by *A. sobria* in patients with neutropenia whose fatal clinical course was just like that described by Martino *et al.*[9] in their report of two cases with acute non-lymphoblastic leukemia who developed septic shock caused by *Aeromonas* species with soft tissue complications. Both studies confirm the potentially aggressive nature of these bacteria in patients with neutropenic cancer. Tsai *et al*. [14] also reported the rapid onset of skin necrosis and sepsis caused by *A. sobria* in two patients with diabetes who died only a few days after admission. Finally, Stano *et al.*[4] described a fatal *A. sobria* sepsis complicated by rhabdomyolysis in a patient with human immunodeficiency virus. What is described in the literature seems to suggest that *A. sobria* is the more pathogenic among *Aeromonas* bacteria since only patients with infections caused by *A. hydrophila* survived [9].

**Table 1:** **Cases of necrotizing fasciitis caused by *Aeromonas *** **species reported in the medical literature**

**Cases**	**Gender/age (year)**	**Predisposing factors**	**Location**	**Species**	**Antibiotics**	**Surgical treatments**	**Outcome/length of stay (days)**
Chang *et al*. [6]	M/60	B-cell acute lymphoblastic leukemia	Lower extremities	*Aeromonas sobria*	Ceftazidime+teicoplanin	None	Death/7
					→imipenem/cilastatin+vancomycin		
	F/39	T-lineage acute lymphoblastic leukemia	Lower extremities	*A. sobria*	Imipenem/cilastatin→levofloxacin	None	Death/3
	M/65	Acute myeloid leukemia	Lower extremities	*A. sobria*	Ceftazidime+teicoplanin	None	Death/5
Tsai *et al*. [14]	M/66	Diabetes mellitus, local wound trauma	Forearm	*A. sobria*	Cefuroxime+gentamicin	Fasciotomy+debridement	Death/2
	M/79	Diabetes mellitus	Lower extremities	*A. sobria*	Vancomycin+ceftazidime	Amputation	Death/11
					→ceftriaxone+metronidazole		
Stano *et al*. [4]	M/43	Human immunodeficiency virus and hepatitis C virus infected	Lower extremities (rhabdomyolysis)	*A. sobria*	Metronidazole+ceftriaxone	None	Death/-
Martino *et al*. [9]	F/41	Acute non-lymphoblastic leukemia	Calf (local myonecrosis and pseudoabscess)	*Aeromonas hydrophila*	Ceftazidime+amikacin+ciprofloxacin	Drainage	Cured
	M/50	Acute non-lymphoblastic leukemia	Lower extremities	*A. sobria*	Ceftazidime+amikacin	None	Death/3
Kao *et al*. [16]	M/76	Local trauma	Hand	*Aeromonas schubertii*	Penicillin→minocycline	Fasciotomy+debridement	Death/-
Furusu *et al*. [17]	M/66	Diabetes mellitus, liver dysfunction, hemodialysed	Lower extremity	*A. hydrophila*	Piperacillin+clindamycin	Fasciotomy+debridement→amputation	Cured
					→clindamycin+minocycline		
					+ceftazidime		
					→aztreonam+clindamycin		
Apisarnthanarak *et al*. [18]	M/61	Cirrhosis	Neck soft tissue	*A. hydrophila*	Amoxicillin→ampicillin-clavulanate→ceftriaxone	Debridement	Cured
					+clindamycin+ciprofloxacin		
Minnaganti *et al*. [1]	M/81	Congestive heart failure and peripheral neuropathy	Forearm	*A. hydrophila*	Clindamycin+Levofloxacin	Amputation	Death/-
Kumar *et al*. [19]	M/71	Hypertension and previous coronary artery bypass graft	Lower extremity	*Aeromonas caviae*	Ceftriaxone+amikacin	Fasciotomy+debridement	Cured

M, male; F, female.

Two mechanisms have been proposed to explain the onset of *Aeromonas* soft tissue skin infection. The first mechanism postulates that the bacterium invades through trauma, triggering primary infection of the soft tissue causing subsequent development of sepsis. According to the second mechanism, sepsis is first induced by the pathogen and then by metastatic lesions in the soft tissue [11]. In patients with bacteremia, the source of bacteremia is often unclear. A review reported that *Aeromonas* species are causative organisms more commonly found in those who are exposed to seawater or raw seafood, making fisherman an occupation group at higher risk [3].

The ability of *Aeromonas* to cause disease is affected not only by patient characteristics but also microbial factors, and the products of the organisms are responsible for hypotension, multiorgan failure, and disseminated intravascular coagulation, which may lead to septic shock as a result of vascular leakage and vasodilatation [3].

*A. sobria* is a human pathogen; the cases of bacterial infections described refer to healthy subjects affected with gastrointestinal infection [10]. However, patients with severe wound infection caused by *A. sobria* also develop sepsis and >90% of the patients succumb to their infections; published data have shown that a serine proteinase secreted by *A. sobria*, at infection sites or in the circulation, points to a mechanism of virulence that could be associated with the induction of septic shock caused by infection with this bacterium [10].

Although *Aeromonas* has a propensity to produce at least three β-lactamases, these microorganisms are generally susceptible to the majority of antibiotics active against Gram-negative bacilli. This notwithstanding, increasing antimicrobial resistance among *Aeromonas* species has been observed depending mainly on species, source and country of isolation [4,5]. Some authors recommend obtaining a susceptibility test before deciding the correct treatment regime and delaying proper antibiotics until culture results are available. This delay, however, may endanger patients, especially neutropenic patients [4,6]. After evaluating monomicrobial *Aeromonas,* Ko and Chuang did not observe any major differences in treatment with a combination of aminoglycoside and β-lactam, and they suggested that a broad-spectrum cephalosporin remains one of the main therapeutic choices in invasive *Aeromonas* infections [5].

The two most common pitfalls in management of *Aeromonas* necrotizing fasciitis are the failure to make an early diagnosis and inadequate medical and surgical treatment [6]. The literature reports that early surgical intervention is essential in immunocompromised hosts with this infection [9] and that more aggressive early surgical intervention may be indicated in these patients [14].

In the present case, all predisposing factors for a typical *Aeromonas* infection were present; our patient had a clinical history of hematological disorders and infection was most likely caused by exposure of a wound to contaminated water (the patient had immersed his hands into potentially contaminated water while fishing and was scratched by a catfish). Nevertheless, some aspects contributed to failure of the case. The lack of any clear clinical signs and symptoms prevented early diagnosis. It is crucial to remember that pain which appears to be disproportional to the physical findings is the most consistent manifestation of necrotizing fasciitis, and that the presence of bullae also constitutes an important diagnostic clue. It is suggested in the literature that these two aspects should be taken into account since the earlier necrotizing fasciitis is diagnosed, the better the outcome and the fewer the complications [12]. During the first 36 hours spent in our Medical Department, our patient mainly presented pain and motor disability, making it difficult for the clinicians to formulate a correct diagnosis.

Furthermore, clinical differentiation between *Aeromonas* necrotizing fasciitis and infections caused by other microorganisms is no easy matter. In the case of gas-producing muscular necrosis, as in our patient, an infection of possible *Clostridium* origin should be taken into consideration [15]. In the case of lesions where neither Gram-positive nor anaerobic bacilli are detected, *Aeromonas* ought to be suspected, just as the need to deliver prompt and appropriate empiric antibiotic therapy and aggressive surgical treatment should be considered. Concurrent immunosuppression, consequent to the hematologic disease and the chronic use of steroids may, in our case, have contributed to the pathogenesis and virulence of the *Aeromonas* disease, as well as the unfortunate coexistence of fungal infection which played a key role in the clinical course.

## Conclusions

This is a report of an uncommon case of *Aeromonas sobria* sepsis in an immunocompromised patient. We reviewed the literature and found that the presentation, treatment and final resolution of this case is consistent with the data presented in the literature.

It is, therefore, important for physicians caring for immunocompromised patients with necrotizing fasciitis to consider the possible involvement of *Aeromonas* species as the causative agent of infection. Time is a critical element in the treatment of immunocompromised patients presenting a rapid onset of skin necrosis and progressive sepsis. Early recognition and aggressive medical and surgical therapy are the primary determinants of outcome in the treatment of these patients.

## Consent

Informed written consent was obtained from the patient’s next of kin for publication of this case report and any accompanying images. A copy of the written consent is available for review by the Editor-in-Chief of this journal.

## Abbreviations

CT: Computed tomography; ICU: Intensive care unit; WBC: White blood cell.

## Competing interests

The authors declare that they have no competing interests.

## Authors’ contributions

SS looked after the patient and conceived of the idea for the report and wrote the draft. AB and SS wrote the first draft. CAV and RR revised the manuscript. AR, MVC, SZ and EM looked after the patient and helped to draft the manuscript. All authors read and approved the final manuscript.
